# Supplementary material for: Lrig1- and Wnt-dependent niches dictate segregation of resident immune cells and melanocytes in murine tail epidermis
Source: Development. 2022 Jul 14;149(14):dev200154. doi: 10.1242/dev.200154 (PMC9382897; doi:10.1242/dev.200154)
Supplement: Supplementary information [file develop-149-200154-s1.pdf]

Baess et al. Figure S1

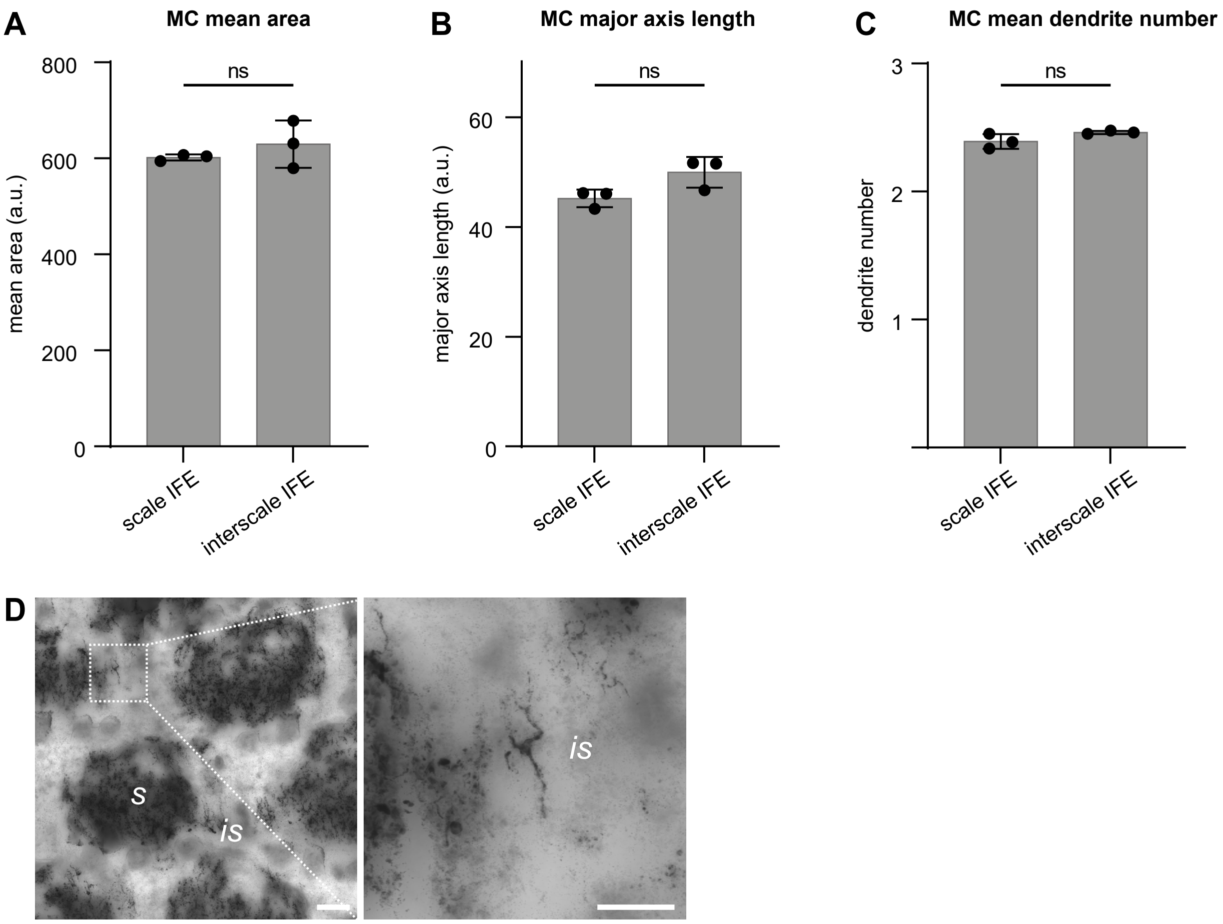

Fig. S1. Interscale IFE contains a small MC population with morphological features similar to their scale-based counterparts and capable of melanin production. (A) Quantification of mean area per MC in 3-months old wild-type C57BL/6 scale and interscale tail IFE. n=3; ns: p=0.7000; mean±s.d.; Mann-Whitney test. (B) Quantification of major axis length per MC in 3-months old C57BL/6 scale and interscale tail IFE. n=3; ns: p=0.1000; mean±s.d.; Mann-Whitney test. (C) Quantification of dendrite numbers per MC in 3-months old C57BL/6 scale and interscale tail IFE. n=3; ns: p=0.2000; mean±s.d.; Mann-Whitney test. (D) Representative images of Fontana-Masson staining in tail epidermis whole-mounts of 3-months old C57BL/6 mice. Representative for n=3. Scale bars: 100 μm (right), 50 μm (left). s: scale, is: interscale.

Baess et al. Figure S2

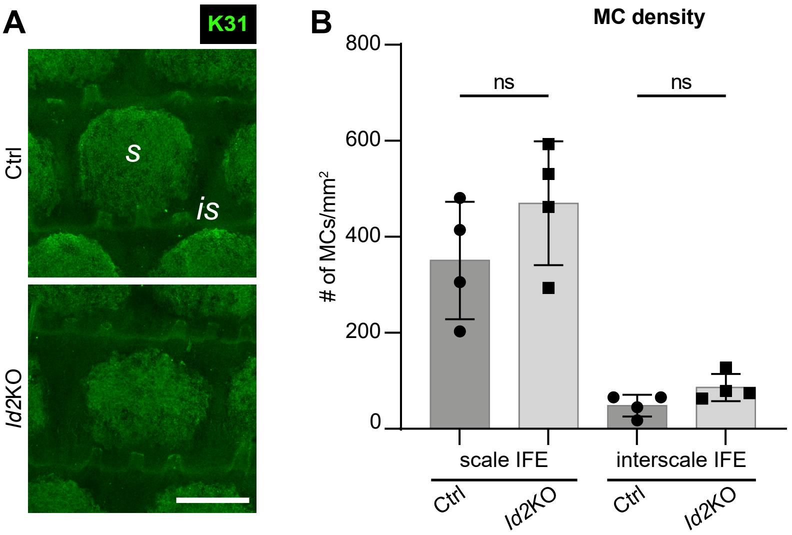

Fig. S2. Epidermal scale differentiation and MC densities are unaffected by deletion of transcription factor Id2. (A) K31 immunostaining of tail epidermis whole-mounts of 3-months old control and *Id2*KO mice. Representative for n=4. Scale bar: 250 µm. (B) Quantification of 3A; MC cell numbers per scale:interscale unit (MCs/mm^2) in tail epidermis from 3-months old control and *Id2*KO mice. n=4; ns: p=0.2962 (scale IFE, Ctrl vs. *Id2*KO); ns: p=0.9343 (interscale IFE, Ctrl vs. *Id2*KO); mean±s.d.; one-way ANOVA/Tukey's multiple comparisons test. s: scale, is: interscale.

Baess et al. Figure S3

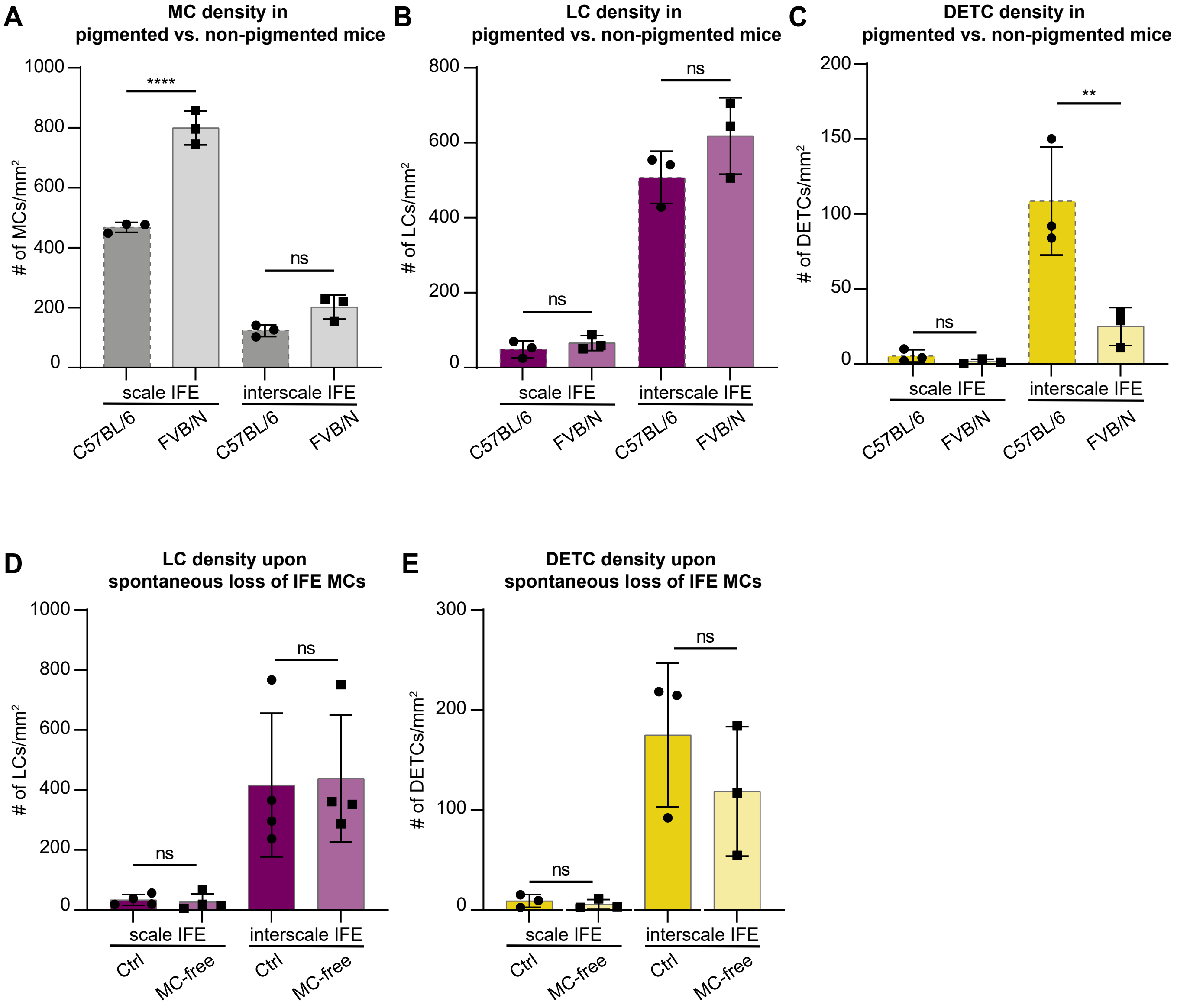

Fig. S3. Epidermis-resident cell types in non-pigmented FVB/N mice and in regions of spontaneous MC loss. (A) Quantification of 4A; MC cell numbers per scale:interscale unit (MCs/mm²) in tail epidermis from 3-months old wild-type C57BL/6 and FVB/N mice. n=3; ****: p<0.0001 (scale IFE, C57BL/6 vs. FVB/N); ns: p=0.1173 (interscale IFE, C57BL/6 vs. FVB/N); mean±s.d.; one-way ANOVA/Tukey's multiple comparisons test. Data for C57BL/6 as shown in Figure 1F. (B) Quantification of 4A; LC cell numbers per scale:interscale unit (LCs/mm²) in tail epidermis from 3-months old wild-type C57BL/6 and FVB/N mice. n=3; ns: p=0.9875 (scale IFE, C57BL/6 vs. FVB/N); ns: p=0.2228 (interscale IFE, C57BL/6 vs. FVB/N); mean±s.d.; one-way ANOVA/Tukey's multiple comparisons test. Data for C57BL/6 as shown in Figure 1G. (C) Quantification of 4B; DETC cell numbers per scale:interscale unit (DETCs/mm²) in tail epidermis from 3-months old wild-type C57BL/6 and FVB/N mice. n=3; ns: p=0.9944 (scale IFE, C57BL/6 vs. FVB/N); **: p=0.0032 (interscale IFE, C57BL/6 vs. FVB/N); mean±s.d.; one-way ANOVA/Tukey's multiple comparisons test. Data for C57BL/6 as shown in Figure 1H. (D) Quantification of 4G; LC cell numbers per scale:interscale unit (LCs/mm²) in tail epidermis from 3-months old wild-type C57BL/6 mice with spontaneous loss of MCs in posterior tail epidermis. n=4; ns: p>0.9999 (scale IFE, Ctrl vs. MC-free area); ns: p=0.9975 (interscale IFE, Ctrl vs. MC-free area); mean±s.d.; one-way ANOVA/Tukey's multiple comparisons test. (E) Quantification of 4H; DETC cell numbers per scale:interscale unit (DETCs/mm²) in tail epidermis from 3-months old wild-type C57BL/6 mice with spontaneous loss of MCs in posterior tail epidermis. n=3; ns: p=0.9998 (scale IFE, Ctrl vs. MC-free area); ns: p=0.5202 (interscale IFE, Ctrl vs. MC-free area); mean±s.d.; one-way ANOVA/Tukey's multiple comparisons test.

Baess et al. Figure S4

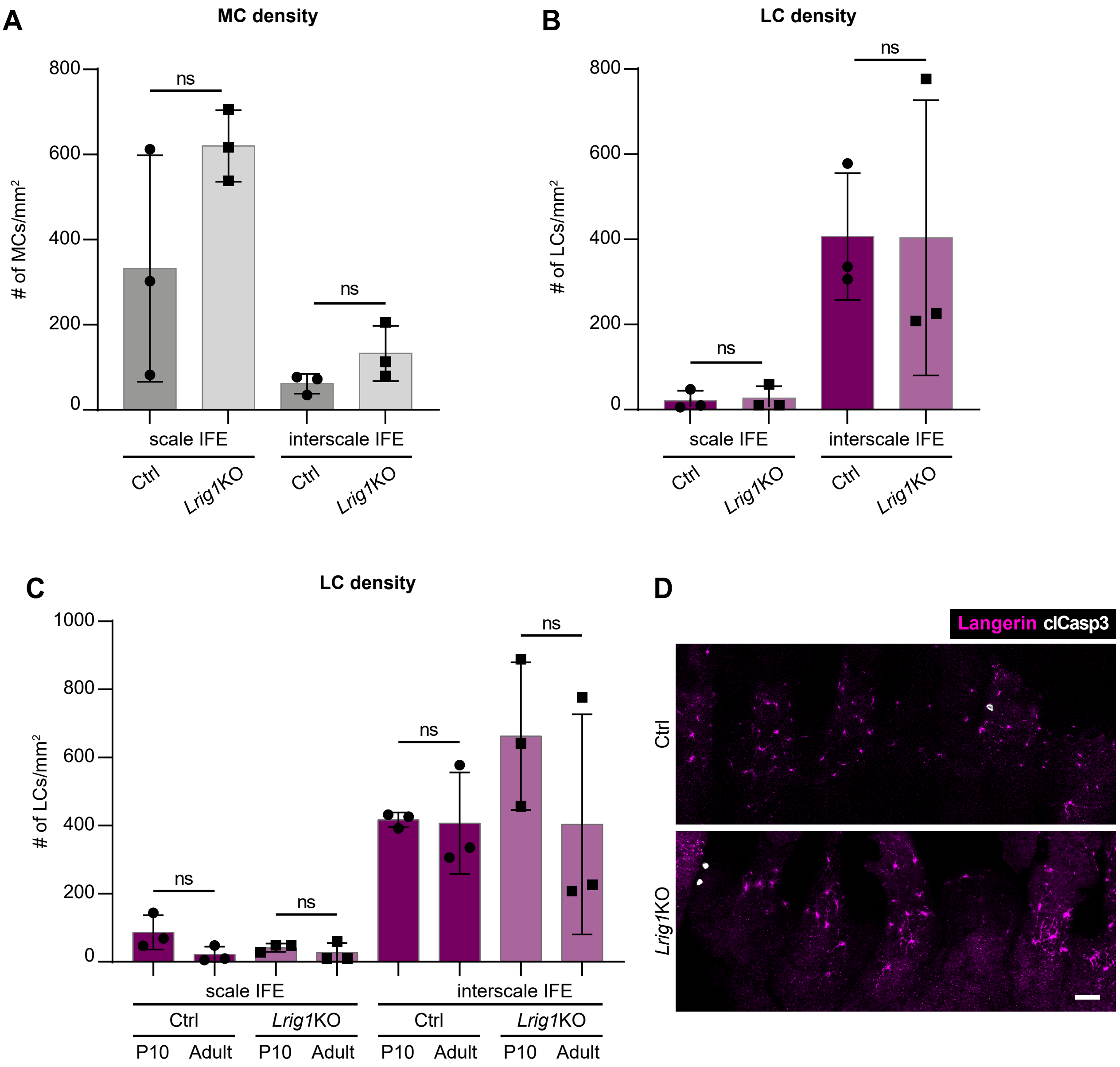

Fig. S4. Analysis of resident cell types in *Lrig1*KO mice. (A) Quantification of 5A; MC cell numbers per scale:interscale unit (MCs/mm²) in tail epidermis from 3-months old control and *Lrig1*KO mice. n=3; ns: p=0.1435 (scale IFE, Ctrl vs. *Lrig1*KO); ns: p=0.9269 (interscale IFE, Ctrl vs. *Lrig1*KO); mean±s.d.; one-way ANOVA/Tukey's multiple comparisons test. (B) Quantification of 5A; LC cell numbers per scale:interscale unit (LCs/mm²) in tail epidermis from 3-months old control and *Lrig1*KO mice. n=3; ns: p>0.9999 (scale IFE, Ctrl vs. *Lrig1*KO); ns: p>0.9999 (interscale IFE, Ctrl vs. *Lrig1*KO); mean±s.d.; one-way ANOVA/Tukey's multiple comparisons test. (C) LC cell numbers per scale:interscale unit (LCs/mm²) in tail epidermis from P10 and 3-months old control and *Lrig1*KO mice (data set of 3-months old mice also shown in B, here compared to younger mice). n=3; ns: p=0.9992 (scale IFE, Ctrl P10 vs. Ctrl Adult); ns: p>0.9999 (scale IFE, *Lrig1*KO P10 vs. *Lrig1*KO Adult); ns: p>0.9999 (interscale IFE, Ctrl P10 vs. Ctrl Adult); ns: p=0.4413 (interscale IFE, *Lrig1*KO P10 vs. *Lrig1*KO Adult); mean±s.d.; one-way ANOVA/Tukey's multiple comparisons test. (D) Langerin and cleaved Caspase3 immunostaining of tail epidermis whole-mounts from 3-months old control and *Lrig1*KO mice. Representative for n=3. Scale bar: 100 µm.

Development • Supplementary information

Baess et al. Figure S5

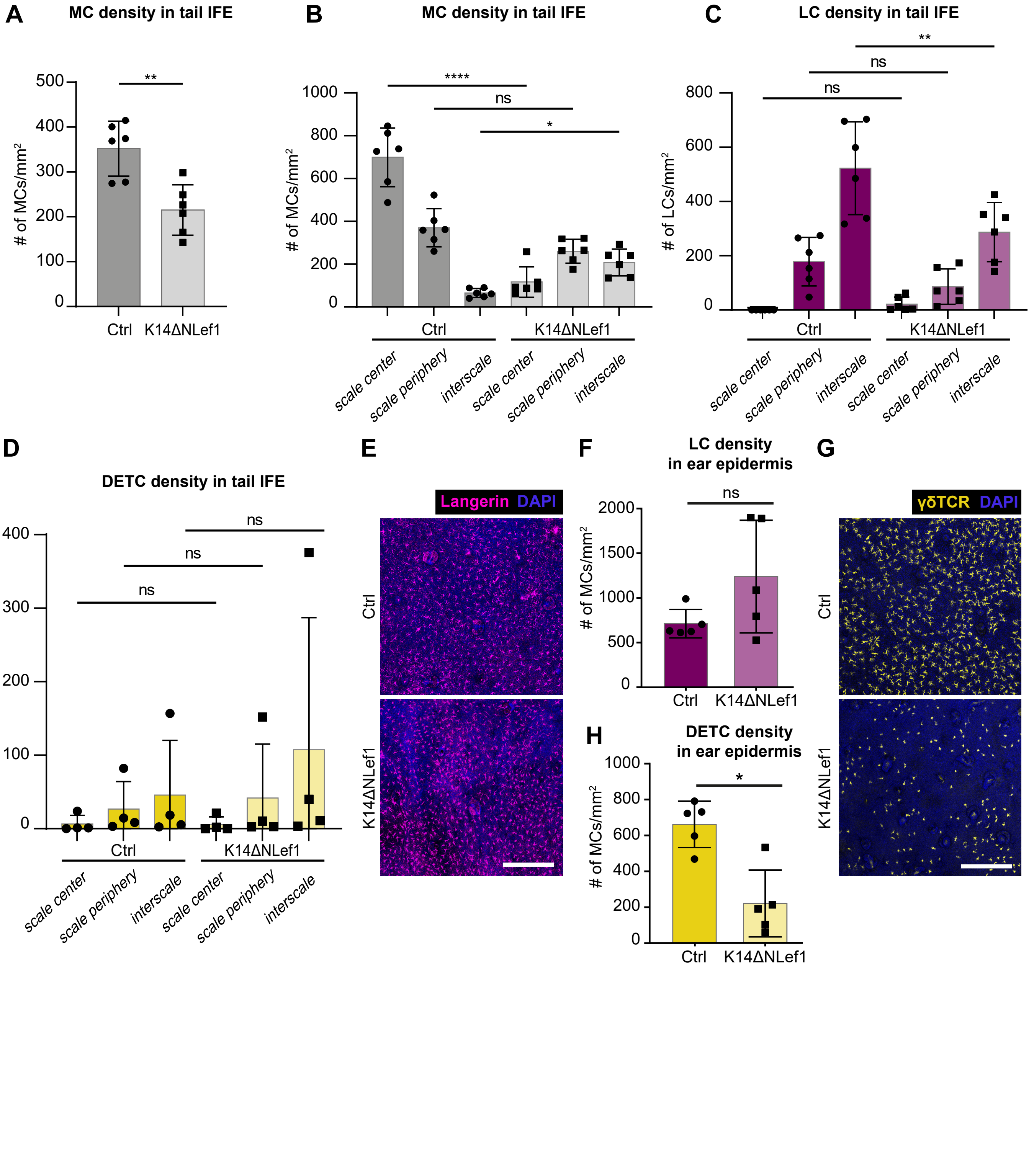

Development • Supplementary information

Fig. S5. Analysis of resident cell types in K14ΔNLef1 tail and ear IFE. (A) Quantification of 6A; MC cell numbers (MCs/mm^2) in tail epidermis from 3-months old control and K14ΔNLef1 mice. n=6; **: p=0.0087; mean±s.d.; Mann-Whitney test. (B) Quantification of 6A; MC cell numbers per scale:interscale unit (MCs/mm²) in tail epidermis from 3-months old control and K14ΔNLef1 mice. n=6; ****: p<0.0001 (scale center, Ctrl vs. K14ΔNLef1); ns: p=0.2070 (scale periphery, Ctrl vs. K14ΔNLef1); *: p=0.0497 (interscale IFE, Ctrl vs. K14ΔNLef1); mean±s.d.; one-way ANOVA/Tukey's multiple comparisons test. (C) Quantification of 6A; LC cell numbers per scale:interscale unit (LCs/mm²) in tail epidermis from 3-months old control and K14ΔNLef1 mice. n=6; ns: p=0.9986 (scale center, Ctrl vs. K14ΔNLef1); ns: p=0.5590 (scale periphery, Ctrl vs. K14ΔNLef1); **: p=0.0021 (interscale IFE, Ctrl vs. K14ΔNLef1); mean±s.d.; one-way ANOVA/Tukey's multiple comparisons test. (D) Quantification of 6B; DETC cell numbers per scale:interscale unit (DETCs/mm²) in tail epidermis from 3-months old control and K14ΔNLef1 mice. n=4; ns: p>0.9999 (scale center, Ctrl vs. K14ΔNLef1); ns: p>0.9999 (scale periphery, Ctrl vs. K14ΔNLef1); ns: p=0.8852 (interscale IFE, Ctrl vs. K14ΔNLef1); mean±s.d.; one-way ANOVA/Tukey's multiple comparisons test. (E) Langerin immunostaining of ear epidermis whole-mounts of 3-months old control and K14ΔNLef1 mice. Nuclei were counterstained using DAPI. Scale bar: 250 μm. (F) Quantification of E; LC densities (number/mm²) in ear epidermis. n=5; ns: p=0.2222; mean ±s.d.; Mann-Whitney test. (G) γδTCR immunostaining of ear epidermis whole-mounts of 3-months old control and K14ΔNLef1 mice. Nuclei were counterstained using DAPI. Scale bar: 250 μm. (H) Quantification of G; DETC densities (number/mm²) in ear epidermis. n=5; *: p=0.0159; mean±s.d.; Mann-Whitney test.

Development • Supplementary information

Baess et al. Figure S6

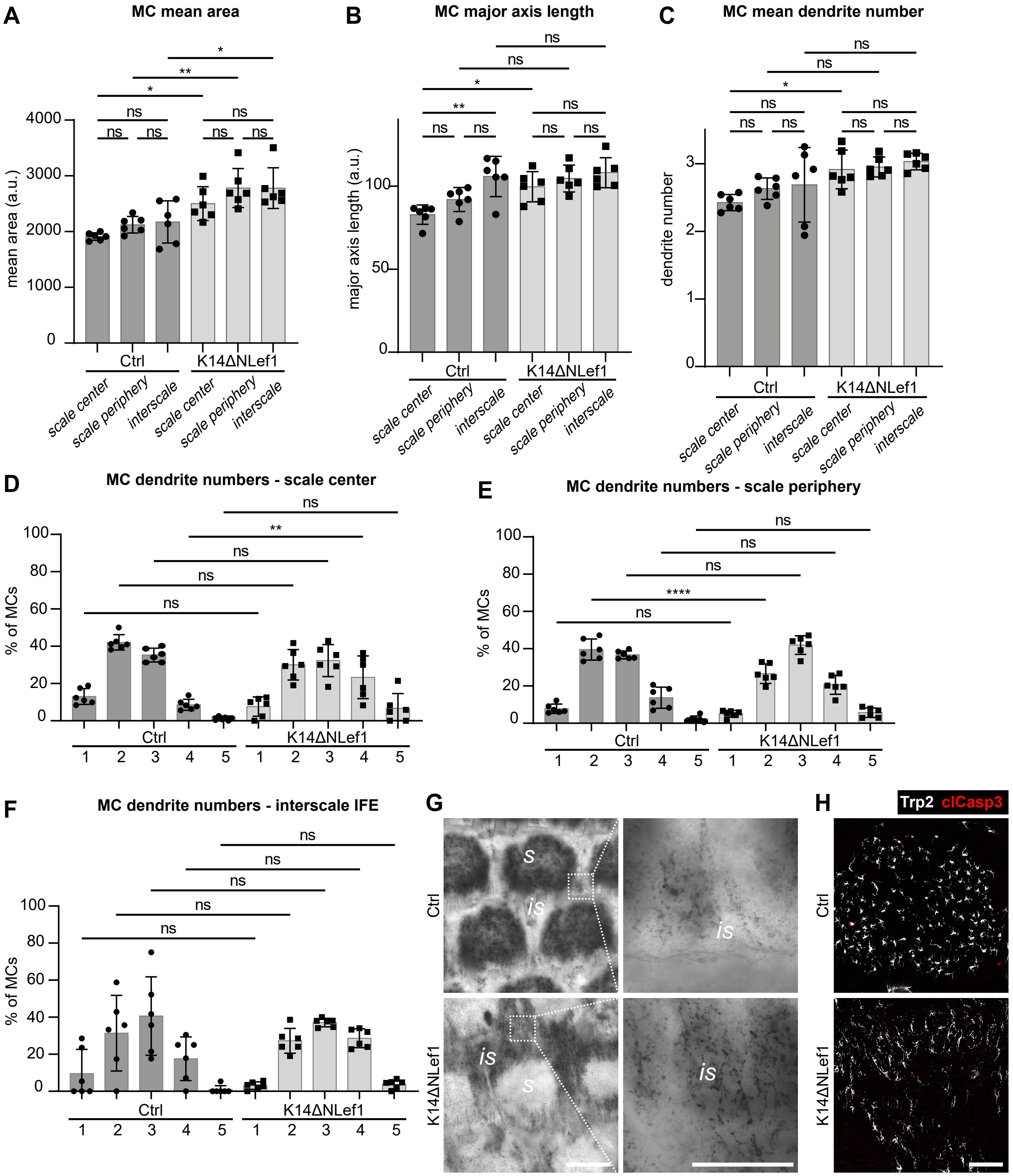

Development • Supplementary information

Fig. S6. Analysis of MC properties in K14ΔNLef1 tail IFE. (A) Quantification of mean area per MC in tail IFE of 3-months old control and K14ΔNLef1 mice. n=6; ns: p=0.8155 (Ctrl, scale center vs. scale periphery); ns: p=0.6484 (Ctrl, scale center vs. interscale IFE); ns: p=0.9997 (Ctrl, scale periphery vs. interscale IFE); ns: p=0.5756 (K14ΔNLef1, scale center vs. scale periphery); ns: p=0.5801 (K14ΔNLef1, scale center vs. interscale IFE); ns: p>0.9999 (K14ΔNLef1, scale periphery vs. interscale IFE); *: p=0.0180 (scale center, Ctrl vs. K14ΔNLef1); **: p=0.0064 (scale periphery, Ctrl vs. K14ΔNLef1); *: p=0.0137 (intersale IFE, Ctrl vs. K14ΔNLef1); mean±s.d.; one-way ANOVA/Tukey's multiple comparisons test. (B) Quantification of major axis length per MC in tail IFE of 3-months old control and K14ΔNLef1 mice. n=6; ns: p=0.4863 (Ctrl, scale center vs. scale periphery); **: p=0.0011 (Ctrl, scale center vs. interscale IFE); ns: p=0.0971 (Ctrl, scale periphery vs. interscale IFE); ns: p=0.9185 (K14ΔNLef1, scale center vs. scale periphery); ns: p=0.5608 (K14ΔNLef1, scale center vs. interscale IFE); ns: p=0.9825 (K14ΔNLef1, scale periphery vs. interscale IFE); *: p=0.0270 (scale center, Ctrl vs. K14ΔNLef1); ns: p=0.1156 (scale periphery, Ctrl vs. K14ΔNLef1); ns: p=0.9975 (intersale IFE, Ctrl vs. K14ΔNLef1); mean±s.d.; one-way ANOVA/Tukey's multiple comparisons test. (C) Quantification of mean dendrite number per MC in tail IFE of 3-months old control and K14ΔNLef1 mice. n=6; ns: p=0.7946 (Ctrl, scale center vs. scale periphery); ns: p=0.5820 (Ctrl, scale center vs. interscale IFE); ns: p=0.9991 (Ctrl, scale periphery vs. interscale IFE); ns: p>0.9999 (K14ΔNLef1, scale center vs. scale periphery); ns: p=0.9765 (K14ΔNLef1, scale center vs. interscale IFE); ns: p=0.9957 (K14ΔNLef1, scale periphery vs. interscale IFE); *: p=0.0489 (scale center, Ctrl vs. K14ΔNLef1); ns: p=0.3645 (scale periphery, Ctrl vs. K14ΔNLef1); ns: p=0.2925 (intersale IFE, Ctrl vs. K14ΔNLef1); mean±s.d.; one-way ANOVA/ Tukey's multiple comparisons test. (D) Quantification of dendrite numbers (% of MCs) in tail IFE scale center compartment of 3-months old control and K14ΔNLef1 mice. n=6; ns: p=0.9083 (1 dendrite, Ctrl vs. K14ΔNLef1); ns: p=0.0635 (2 dendrites, Ctrl vs. K14ΔNLef1); ns: p=0.9983 (3 dendrites, Ctrl vs. K14ΔNLef1); **: p=0.0087 (4 dendrites, Ctrl vs. K14ΔNLef1); ns: p=0.8815 (5 dendrites, Ctrl vs. K14ΔNLef1); mean±s.d.; one-way ANOVA/Tukey's multiple comparisons test. (E) Quantification of dendrite numbers (% of MCs) in tail IFE scale periphery compartment of 3-months old control and K14ΔNLef1 mice. n=6; ns: p=0.9790 (1 dendrite, Ctrl vs. K14ΔNLef1); ****: p<0.0001 (2 dendrites, Ctrl vs. K14ΔNLef1); ns: p=0-4488 (3 dendrites, Ctrl vs. K14ΔNLef1); ns: p=0.1162 (4 dendrites, Ctrl vs. K14ΔNLef1); ns: p=0.9074 (5 dendrites, Ctrl vs. K14ΔNLef1); mean±s.d.; one-way ANOVA/Tukey's multiple comparisons test. (F) Quantification of dendrite numbers (% of MCs) in tail IFE interscale compartment of 3-months old control and K14ΔNLef1 mice. n=6; ns: p=0.9910 (1 dendrite, Ctrl vs. K14ΔNLef1); ns: p=0.9998 (2 dendrites, Ctrl vs. K14ΔNLef1); ns: p>0.9999 (3 dendrites, Ctrl vs. K14ΔNLef1); ns: p=0.7885 (4 dendrites, Ctrl vs. K14ΔNLef1); ns: p>0.9999 (5 dendrites, Ctrl vs. K14ΔNLef1); mean±s.d.; one-way ANOVA/Tukey's multiple comparisons test. (G) Representative micrographs of Fontana-Masson staining in tail epidermis whole-mounts of 3-months old Ctrl and K14ΔNLef1 mice. Representative for n=3. Scale bars: 300 µm (left), 100 µm (right). (H) Langerin and cleaved Caspase3 immunostaining of tail epidermis whole-mounts from 3-months old control and K14ΔNLef1 mice. Representative for n=3. Scale bar: 100 µm. s: scale, is: interscale.

Table S1. Antibodies and imaging reagents used in this study

**Primary antibodies**				
**antibody (supplier)**	**catalog nr.**	**clone/Ref.**	**species**	**dilution**
K31 (Progen)	GP-HHA1	polyclonal	guinea pig	IF 1:100
Trp2 (Santa Cruz Biotechnology)	sc-10451	polyclonal	goat	IF 1:100
Langerin (Invitrogen)	14-2073-80	eBioRMUL.2	rat	IF 1:100
MHC II (M. Pasparakis lab, CECAD, Cologne)	-	-	rat	IF 1:100
CD45 (Invitrogen)	14-0451-82	30-F11	rat	IF 1:100
Cleaved caspase 3 (R&D Systems)	AF835	-	rabbit	IF 1:100
**Secondary antibodies**				
Goat anti-guinea pig AF488 (Invitrogen)	A11073	polyclonal	goat	IF 1:500
Donkey anti-rat AF594 (Dianova)	712-585-153	polyclonal	donkey	IF 1:500
Donkey anti-goat AF647 (Invitrogen)	A21447	polyclonal	donkey	IF 1:500
Goat anti-rat AF647 (Invitrogen)	A21247	polyclonal	goat	IF 1:500
Goat anti-rabbit AF568 (Invitrogen)	A11036	polyclonal	goat	IF 1:500
Donkey anti-goat AF594 (Invitrogen)	A11058	polyclonal	donkey	IF 1:500
Donkey anti-rabbit AF488 (Invitrogen)	A21206	polyclonal	donkey	IF 1:500
Goat anti-guinea pig AF568 (Invitrogen)	A11075	polyclonal	goat	IF 1:500
**Other reagents**				
DAPI (Carl Roth)	-	-	-	IF 1:500
Fontana-Masson staining kit (Sigma-Aldrich)	HT200	-	-	-
**Directly-conjugated antibodies**				
FITC anti-mouse TCR γ/δ (BioLegend)	118105	GL3	Armenian hamster	IF 1:300

Development • Supplementary information
