# Supplementary material for: Lrig1- and Wnt-dependent niches dictate segregation of resident immune cells and melanocytes in murine tail epidermis
Source: Development. 2022 Jul 14;149(14):dev200154. doi: 10.1242/dev.200154 (PMC9382897; doi:10.1242/dev.200154)
Supplement: Reviewer comments [file develop-149-200154_review_history.pdf]

Lrig1- and Wnt-dependent niches dictate segregation of resident immune cells and melanocytes in murine tail epidermis

Susanne C. Baess, Ann-Kathrin Burkhart, Sabrina Cappello, Annika Graband, Kristin Seré, Martin Zenke, Catherin Niemann and Sandra Iden
DOI: 10.1242/dev.200154

Editor: Paul Martin

Review timeline

Original submission:	31 August 2021
Editorial decision:	29 November 2021
First revision received:	8 March 2022
Editorial decision:	19 April 2022
Second revision received:	24 May 2022
Accepted:	13 June 2022

Original submission

First decision letter

MS ID#: DEVELOP/2021/200154

MS TITLE: Lrig1 and Wnt signaling instruct segregation of resident immune cells and melanocytes into distinct epidermal niches

AUTHORS: Susanne C. Baess, Annika Graband, Kristin Sere, Martin Zenke, Catherin Niemann, and Sandra Iden

I have now received all the referees' reports on the above manuscript, and have reached a decision. The referees' comments are appended below, or you can access them online: please go to BenchPress and click on the 'Manuscripts with Decisions' queue in the Author Area.

As you will see, the referees express considerable interest in your work, but have some significant criticisms and recommend a substantial revision of your manuscript before we can consider publication. If you are able to revise the manuscript along the lines suggested, which I think will involve further experiments/analysis, I will be happy receive a revised version of the manuscript. Your revised paper will be re-reviewed by one or more of the original referees, and acceptance of your manuscript will depend on your addressing satisfactorily the reviewers' major concerns. Please also note that Development will normally permit only one round of major revision.

We are aware that you may be experiencing disruption to the normal running of your lab that make experimental revisions challenging. If it would be helpful, we encourage you to contact us to discuss your revision in greater detail. Please send us a point-by-point response indicating where you are able to address concerns raised (either experimentally or by changes to the text) and where you will not be able to do so within the normal timeframe of a revision. We will then provide further guidance. Please also note that we are happy to extend revision timeframes as necessary.

Please attend to all of the reviewers' comments and ensure that you clearly highlight all changes made in the revised manuscript. Please avoid using 'Tracked changes' in Word files as these are lost in PDF conversion. I should be grateful if you would also provide a point-by-point response detailing

© 2022. Published by The Company of Biologists under the terms of the Creative Commons Attribution License (https://creativecommons.org/licenses/by/4.0/).

how you have dealt with the points raised by the reviewers in the 'Response to Reviewers' box. If you do not agree with any of their criticisms or suggestions please explain clearly why this is so.

Reviewer 1

Advance summary and potential significance to field

General comments:

This manuscript details the location of non-epidermal cells (i.e. Langerhans, DETC and melanocyte cells) within the distinct compartments of scale and non-scale forming epidermis in the mouse tail skin. Moreover, the authors show that the signals within the epidermis influencer patterning of melanocytes in the tail.
Other works (notably Park et al, 2021 NCB and others) have shown that the location of skin-immune cells is not random in other skin tissues, but this manuscript goes beyond this previous study to detail the parallel influences directing skin-immune cells and melanocytes in the tail. There could, and probably should, be additional integration of the findings presented here integrate with previous works, however overall this manuscripts presents novel findings that have the potential to be interesting to a wide audience.

Comments for the author

Major concerns:
1) The title overstates the findings. Previous publications show that LRIG1 and Wnt signaling influence the epidermal specification of the scale and non-scale regions of the tail and this work demonstrates that regional differences in the epidermis influence patterning of non-keratinocyte cells. However, how LRIG 1/Wnt signaling “instructs” immune and melanocyte patterning remains unclear.
2) Antibody detection of Langerin/gdTCR/TRP2 shows that some TRP2+ MCs localise in the non-scale forming regions adjacent to skin-immune cells (LCs and DETCs). However, the distribution of these cells types is not fully segregated, as was suggested in the manuscript, and this should be corrected and discussed.
Additional, finer scale quantification of the location of MCs in the non-scale epidermis may reveal further patterning of these MCs in the tail.
3) In the K14 deltaNLEF1 tail skin, it is not clear the impact disruption of Wnt-Lef1 signaling in all regions of the tail epidermis. K14 will be expressed in all basal cells, therefore the impact of the K14deltaNLEF1 transgene on the non-scale epidermis should be discussed. This is important as melanocytes seem to be driven out of the K31 plaques in K14deltaNLEF1 tails but the is not clear melanocyte re-distribution to the non-scale sites is due to the scale region becoming inhospitable or whether the melanocytes are being actively recruited to inter-scale regions? Do the actual number of melanocytes per tissue area change?
What about melanocyte viability?

Reviewer 2

Advance summary and potential significance to field

In this manuscript, Baess et al., describe the localization of immune and melanocyte populations in murine tail skin, as well as investigate some of the central pathways that may be involved in maintaining these distributions. The authors begin their paper first by carefully describing inter-scale vs scale epidermal compartments, validating previous work in the literature (Schweizer&Marks, 1977; Glover et al., 2015; Gomez et al., 2013). They then go on to show that melanocyte (MC) and immune cells (LCs and DETCs) localize independently to each other in their respective epidermal departments. Finally, the authors show MC:immune cell localization is in part Wnt-Lef1-dependent.

© 2022. Published by The Company of Biologists under the terms of the Creative Commons Attribution License (https://creativecommons.org/licenses/by/4.0/).

Comments for the author

While the manuscript is well written and potentially tackles an interesting area of research, it remains in its current form incomplete. The authors, should try to further their study by either providing a physiological consequences to MC:immune cell partitioning, or further investigate the molecular mechanism that allow MC:immune cells to localize in different epidermal compartments.

Major Comments:
1) In Figure 1, the authors nicely characterize the MC:immune localization in scale and interscale tissue.
• While the images are nicely done, MC:immune localizations should be quantified: cell #s/area in the scale vs interscale locations should be performed at steady state and in the time-course from P5-P21.
• Where are the DETCs localized at P5/P10/P21?
• Figure 1A: a fraction of MCs appear also present in the interscale region. Is this a different population of MCs? Other markers should be explored to determine if MC populations are heterogenous in the tail skin, and if this heterogeneity is driven by anatomical positioning (especially harnessing KO animals from Figures 3 and 4).
2) Similarly, in Figure 2A, MCs numbers appear different in Id2KO animals, and the MC-network appears less dense. MCs numbers/area should be provided in WT and Id2KO conditions. Immune cells #s/area should similarly be quantified in the interscale regions in FVB/N and MC-free animals. In addition, loss of DETCs should also be assessed in Id2KO and FVB/N animals. These quantifications are critical as numbers of MC/immune cells appear different in these knock-out conditions suggesting that while positioning of MC/immune cells may be independent of each other- total numbers of cells may be affected, changing the interpretations of these results.
3) In Figures 3B, the authors state that “MCs assumed a band-like expansion corresponding to the K31-labelled scale band, whereas LCs/DETCs were restricted to the is-stripe”. What happens to LCs/DETCs between P10 and 3-mo animals: P10 animals show LC localization in small interscale regions perpendicular to the HFs that are absent in 3-mo animals. Are they actively pushed out/displaced? Do they die? A kinetic time course should be provided on what is happening as these cells as they are re-localizing/displaced/dyeing.
4) Using the K14-Lef1ko animals, numbers of MCs/area should be provided for each of the three regions in addition to %s.
More importantly, what are the functional/physiological consequences to MC biology now that they localize to the interscale region? Do they change their ability to perform critical MC functions? Do they increase their “immune-surveillance” functions? Laser capture experiments/Spatial transcriptomics/utilization of alternate marker expression with subsequent sorting/sequencing could be used to tease some of the questions apart.
Thus, while this manuscript is nicely written and discusses an interesting area of skin biology, mechanistic/functional experiments would be needed to be generate a manuscript of broader interest to the Development readership.

First revision

Author response to reviewers' comments

Reviewer 1 Advance Summary and Potential Significance to Field:

General comments:

This manuscript details the location of non-epidermal cells (i.e. Langerhans, DETC and melanocyte cells) within the distinct compartments of scale and non-scale forming epidermis in the mouse tail skin. Moreover, the authors show that the signals within the epidermis influencer patterning of melanocytes in the tail. Other works (notably Park et al, 2021 NCB and others) have shown that the location of skin-immune cells is not random in other skin tissues, but this manuscript goes beyond this previous study to detail the parallel influences directing skin- immune cells and melanocytes in the tail. There could, and probably should, be additional integration of the findings presented here integrate with previous works, however, overall this manuscripts

© 2022. Published by The Company of Biologists under the terms of the Creative Commons Attribution License (https://creativecommons.org/licenses/by/4.0/).

presents novel findings that have the potential to be interesting to a wide audience.

For better integration of our data with previous work, we now cite the work by Park et al., NCB 2021, in the introduction (revised **introduction, second paragraph**, page 4), and included a new point on this in the discussion (revised **discussion, second paragraph**, page 9).

Reviewer 1 Comments for the Author:

Major concerns:

1) *The title overstates the findings. Previous publications show that LRIG1 and Wnt signaling influence the epidermal specification of the scale and non-scale regions of the tail and this work demonstrates that regional differences in the epidermis influence patterning of non-keratinocyte cells. However, how LRIG 1/Wnt signaling "instructs" immune and melanocyte patterning remains unclear.*

We thank this reviewer for very constructive and helpful feedback on our original manuscript.
We modified the title as follows: "Lrig1 and Wnt dependent niches dictate segregation of resident immune cells and melanocytes in murine tail epidermis".

2) *Antibody detection of Langerin/gdTCR/TRP2 shows that some TRP2+ MCs localise in the non-scale forming regions adjacent to skin-immune cells (LCs and DETCs). However, the distribution of these cells types is not fully segregated, as was suggested in the manuscript, and this should be corrected and discussed.*
Additional, finer scale quantification of the location of MCs in the non-scale epidermis may reveal further patterning of these MCs in the tail.

Thank you for this important comment. Indeed, a small population of MCs is detected in the interscale compartment, which has also been reported by others previously (Glover et al., 2015; Köhler et al., 2017). To characterize the interscale-based MCs in our study, we carried out quantitative analyses of MC (plus LC and DETC) populations regarding their numbers and distribution. The new data depict the large MC population in scale IFE and a small MC population in interscale IFE, respectively (revised **Fig. 1C, F**). In search of potential differences between scale- and interscale-residing MCs we performed different biochemical and morphometric analyses. CellProfiler-assisted automated image analysis revealed similar morphological features of both MC populations regarding mean cell area, number of dendrites, and dendritic length (revised **Fig. S1A-C**). Moreover, by Fontana-Masson staining (visualizing argentaffinic substances, i.e. melanin) we detected abundant melanin levels in interscale MCs (revised **Fig. S1D**). No further significant patterning of interscale MCs within this compartment could be noted, perhaps due to the small size of this population.

Together, this refined analysis of MCs in scale and interscale IFE provides insight into their distribution, morphology and hallmarks of MC differentiation (i.e. melanin production). We mention and discuss these two populations in the revised manuscript (**results, first section,** page 5; **discussion, last paragraph,** page 10).

3) *In the K14 deltaNLEF1 tail skin, it is not clear the impact disruption of Wnt-Lef1 signaling in all regions of the tail epidermis. K14 will be expressed in all basal cells, therefore the impact of the K14deltaNLEF1 transgene on the non- scale epidermis should be discussed. This is important as melanocytes seem to be driven out of the K31 plaques in K14deltaNLEF1 tails but the is not clear melanocyte re-distribution to the non-scale sites is due to the scale region becoming inhospitable or whether the melanocytes are being actively recruited to inter-scale regions?*

This is indeed an interesting question. Gomez et al. (2013; PMID: 24052938, their Fig. 4A) previously reported that endogenous Lef1 is expressed in scale IFE and not detectable in interscale IFE. We thus expect that transgenic ΔNLef1, though expressed throughout the basal IFE in all compartments, predominantly inhibits Lef1 function in scale regions. In

© 2022. Published by The Company of Biologists under the terms of the Creative Commons Attribution License (https://creativecommons.org/licenses/by/4.0/).

that case, we would conclude that inhibiting Wnt-Lef1 function renders the scale IFE inhospitable for MCs. Yet, we agree with this reviewer that effects of ΔNLef1 expression in interscale regions cannot be formally excluded, potentially attracting MCs to this area. Therefore, we adapted our discussions on this aspect in the manuscript (revised **discussion,** end of **second paragraph,** page 9).

Do the actual number of melanocytes per tissue area change?

We analyzed the number of melanocytes per tissue area in control and mutant mice. In line with the original data of altered distribution of MCs in K14ΔNLef1 mutant mice (revised Fig. 6D), we detected a significant reduction of MC numbers in the scale and a significant increase of MC numbers in the interscale area (revised **Fig. S5B**). Notably, not only the numbers per scale (or interscale) were altered but also the total number of MCs in the IFE of K14ΔNLef1 mutant mice was reduced (revised **Fig. S5A**).

What about melanocyte viability?

To assess the viability of MCs in K14ΔNLef1 mutant mice, we performed immunostainings for cleaved Caspase3. While single apoptotic cells in hair follicles and a few mutant keratinocytes in the IFE could be detected, we did not observe apoptotic MCs, neither in scale nor in interscale regions (revised **Fig. S6H**). These results suggest that changes in MC viability do not predominantly account for the altered localization of MCs in K14ΔNLef1 IFE.
Moreover, similar to the new quantifications done for control tissues (see point 2), we conducted automated morphometric analyses of MCs in K14ΔNLef1 tissues in the different compartments. We noted a significant increase of MC area in the scale center, periphery, and interscale regions in mutant mice (revised **Fig. S6A**). Moreover, K14ΔNLef1 MCs residing in the scale center showed a significant increase of number of dendrites and mean axis length (revised **Fig. S6B-F**), indicative of differentiated MC morphology. While the underlying causes for these morphological changes in K14ΔNLef1 MCs remain open, a likely contributing factor could be the overall reduction of MC numbers (and hence network density), potentially leading to elongation of individual MCs and their dendrites to compensate for the decline in MCs.

Reviewer 2 Advance Summary and Potential Significance to Field:

In this manuscript, Baess et al., describe the localization of immune and melanocyte populations in murine tail skin, as well as investigate some of the central pathways that may be involved in maintaining these distributions. The authors begin their paper first by carefully describing inter-scale vs scale epidermal compartments, validating previous work in the literature (Schweizer&Marks, 1977; Glover et al., 2015; Gomez et al., 2013). They then go on to show that melanocyte (MC) and immune cells (LCs and DETCs) localize independently to each other in their respective epidermal departments. Finally, the authors show MC:immune cell localization is in part Wnt-Lef1-dependent.

Reviewer 2 Comments for the Author:

While the manuscript is well written and potentially tackles an interesting area of research, it remains in its current form incomplete. The authors, should try to further their study by either providing a physiological consequences to MC:immune cell partitioning, or further investigate the molecular mechanism that allow MC:immune cells to localize in different epidermal compartments.

Major Comments:

1) *In Figure 1, the authors nicely characterize the MC:immune localization in scale and interscale tissue.*
- *While the images are nicely done, MC:immune localizations should be quantified: cell #s/area in*

© 2022. Published by The Company of Biologists under the terms of the Creative Commons Attribution License (https://creativecommons.org/licenses/by/4.0/).

the scale vs interscale locations should be performed at steady state and in the time-course from P5-P21.

We wish to thank this reviewer for very constructive feedback, which helped to improve this manuscript. We have now consistently quantified MCs, LCs and DETCs to provide detailed information on these epidermis- resident cell types in developmental as well as adult stages (revised **Fig. 1C-H**, **Fig. 2C-F**, and **Fig. S1A-C**).

• *Where are the DETCs localized at P5/P10/P21?*

DETCs colocalize with LCs early on in the future interscale compartment (revised **Fig. 2B**, **Fig. 5F**). We also quantified the densities of each cell type studied, demonstrating not only the significant growth of epidermal scale:interscale units during postnatal skin development (revised **Fig. 2F**) but also the concomitant non-linear expansion of LCs and DETCs (revised **Fig. 2D,E**) upon postnatal proliferative burst.

• *Figure 1A: a fraction of MCs appear also present in the interscale region. Is this a different population of MCs? Other markers should be explored to determine if MC populations are heterogenous in the tail skin, and if this heterogeneity is driven by anatomical positioning (especially harnessing KO animals from Figures 3 and 4).*

Thank you for raising this interesting point. Indeed, there is a small fraction of MCs localizing to interscale IFE, in line with previous reports by Glover et al. (2015) and Köhler et al. (2017). We now provide quantitative data of MC (and LC and DETC) populations in the different compartments (MCs: revised **Fig. 1C,F**). To investigate a potential heterogeneity of MCs in tail skin, we performed different molecular and morphometric analyses. Hallmarks of functional, differentiated MCs are melanin production and dendritic morphology. The morphometric analyses did not reveal differences between scale and interscale MCs regarding MC mean area, number of dendrites, and dendritic length (revised **Fig. S1A-C**). Moreover, employing Fontana-Masson staining (visualizing argentaffinic substances, i.e. melanin) we were able to detect abundant melanin levels both in scale and interscale MCs (revised **Fig. S1D**). Together, these additional experiments did not indicate obvious heterogeneity of MCs residing in the scale vs. interscale IFE of control mice. Further analyses beyond the scope of this manuscript will be required to learn if the very small interscale MC fraction can be distinguished from the main scale MC population by e.g. more specific transcriptional signatures. In the revised manuscript we now mention and discuss these two different locations of MCs in tail IFE (**results, first section**, page 5, and **discussion, last paragraph**, page 10).

Although we did not obtain signs of heterogeneity in scale vs. interscale MCs in control mice, we went on to analyze different MC morphological and functional parameters in K14ΔNLef1 mice. We detected a significant increase of MC area in the scale center, periphery, and interscale regions in mutant mice, thus independent of anatomical positioning (revised **Fig. S6A**). Moreover, K14ΔNLef1 MCs residing in the scale center showed a significant increase of number of dendrites and mean axis length, indicative of differentiated MC morphology (revised **Fig. S6B-F**). The exact causes of these morphological changes in K14ΔNLef1MCs remain open; however, a likely contributing factor could be the overall reduction of MC numbers (and hence network density), potentially leading to elongation of individual MCs and their dendrites to compensate for the decline in MCs.

2) *Similarly, in Figure 2A, MCs numbers appear different in Id2KO animals, and the MC-network appears less dense. MCs numbers/area should be provided in WT and* ***Id2KO*** *conditions. Immune cells #s/area should similarly be quantified in the interscale regions in FVB/N and MC-free animals. In addition, loss of DETCs should also be assessed in Id2KO and FVB/N animals. These quantifications are critical as numbers of MC/immune cells appear different in these knock- out conditions suggesting that while positioning of MC/immune cells may be independent of each other- total numbers of cells may be affected, changing the interpretations of these results.*

© 2022. Published by The Company of Biologists under the terms of the Creative Commons Attribution License (https://creativecommons.org/licenses/by/4.0/).

We now quantified the numbers of MCs in WT and Id2KO conditions, revealing no differences in MC numbers per area in Id2KO mice (revised **Fig. S2B**). Moreover, we demonstrated the expected absence of DETCs in the Id2KO model (revised **Fig. 3C**), in line with a requirement of Id2 for LCs and DETCs reported previously (Hacker et al., 2003; Seré et al., 2012; Yokota et al., 1999).

Regarding the models of altered pigmentation, we further assessed and quantified MCs, LCs and DETCs in FVB/N vs. C57BL/6 mice (revised **Fig. 4A-E**) and upon spontaneous loss of MCs (revised **Fig. 4F-J**). Complementing the original data for MC and LC positioning, we now provide quantitative data on DETC distribution in scale vs. interscale regions, demonstrating that DETC distribution is similar in FVB/N vs. C57BL/6, with a profound enrichment in the interscale region as seen for LCs (revised **Fig. 4B,E**). Similarly, the distribution of DETCs is unaffected by spontaneous MC loss, again as the case for LCs (revised **Fig. 4J**). These data support our original conclusion that the positioning of IFE-residing immune cells is independent of melanin production and of presence of MCs. Yet, we fully agree with the reviewer that beyond their distribution the total numbers of these cell types could be affected by changes in MC functions. Our quantitative analyses indeed revealed partial alterations in this regard. FVB/N mice, in which MCs are unable to produce melanin due to a point mutation in the tyrosinase gene (PMID 2124349), showed a higher number of MCs in the scale IFE compared to C57BL/6 mice (revised **Fig. S3A**). This was associated with a significant downregulation of DETCs in the adjacent interscale region (revised **Fig. S3C**). However, there was no significant reduction of DETC numbers in the independent model of spontaneous MC loss (revised **Fig. S3E**). We therefore suspect that this difference in DETC numbers in the albino vs. pigmented strains is due to additional allele variations that exist between the FVB/N and C57BL/6 strains, a matter that remains further clarification beyond this work.

3) *In Figures 3B, the authors state that “MCs assumed a band-like expansion corresponding to the K31-labelled scale band, whereas LCs/DETCs were restricted to the is-stripe”. What happens to LCs/DETCs between P10 and 3-mo animals: P10 animals show LC localization in small interscale regions perpendicular to the HFs that are absent in 3-mo animals. Are they actively pushed out/displaced? Do they die? A kinetic time course should be provided on what is happening as these cells as they are re-localizing/displaced/dyeing.*

This is an intriguing question though difficult to address given the slow scale fusion process and the low numbers of LCs and DETCs. Moreover, due to our recent laboratory move (change of institutions) and limited capacities of the involved animal facilities in pandemic times to perform the necessary strain rederivation, at present we do not have the *Lrig1*-KO mouse line available as live stock. Unfortunately, also our collaborator from whom we obtained these mice initially does not keep them anymore, nor do other laboratories that we approached. We were therefore restricted to tissues that we had collected during this study to at least partly address above questions.

With the tissues available we quantified MC and LC distribution in *Lrig1*-KO mice, confirming enrichment of MCs to scale and of LCs to interscale IFE, respectively (revised **Fig. 5A,C,D**). Similarly, the numbers of LCs and MCs per area (density) reflect such enrichment in these areas (revised **Fig. S4A,B**). Interestingly, despite the smaller size of interscale regions in adult *Lrig1*-KO mice, LC density in interscale regions was comparable to that of control mice (revised **Fig. S4B**). More importantly, comparing interscale-localized LC numbers of P10 and adult *Lrig1*-KO mice revealed that the LC network density was kept largely stable during scale fusion (revised **Fig. S4C**, and revised **discussion**, **second paragraph**, page 9), further arguing against LCs being pushed into the shrinking interscale region. Together, these data suggest that LCs are either lost or that their expansion is compromised in the process of scale fusion in *Lrig1*-KO mice. To assess potential underlying mechanisms, we performed cleaved Caspase 3 immunostainings in control and *Lrig1*-KO tissues. While internal controls verified successful immunostaining we could not identify apoptotic immune cells in interscale regions or elsewhere (revised **Fig. S4D**). Although these analyses cannot cover the entire, potentially complex, MC-immune cell redistribution, the obtained data to not provide evidence that apoptosis is a dominant mechanism through which LCs are lost in the course of scale fusion. Future studies using

© 2022. Published by The Company of Biologists under the terms of the Creative Commons Attribution License (https://creativecommons.org/licenses/by/4.0/).

mice with genetically labelled LC populations, combined with *Lrig1*-KO, will be required to understand the full dynamics and cellular mechanisms through which LC densities are kept stable during scale fusion events *in vivo*.

4) *Using the K14-Lef1ko animals, numbers of MCs/area should be provided for each of the three regions in addition to %s.*

The requested numbers of MCs per area (density) are now provided in revised **Fig. S5A,B**. The results show a significant reduction of MC numbers/area in the scale region and significant increase of MC numbers/area in the interscale region of K14ΔNLef1 mice compared to controls (revised **Fig. S5B**). This is in line with the distribution (%) data in tail skin (revised **Fig. 6A-D**) and the increase of numbers of MCs/area in ear epidermis (revised **Fig. 6E,F**) shown in the original version of the manuscript. Interestingly though, next to this remarkable shift in MC localization pattern, our quantitative analysis further revealed that in K14ΔNLef animals the total number of MCs is reduced in tail skin IFE (revised **Fig. S5A,B**), i.e. the strong reduction of scale MCs is not fully compensated by the increase in the interscale MC population. The causes for this MC reduction in tail IFE are at present unknown; however, we did not observe increased apoptosis of MCs in mutant mice (revised **Fig. S6H**).

Additionally, we now provide quantitative data for the distribution (%; revised **Fig. 6G,H**) and densities (#/area; revised **Fig. S5C,D**) of LCs and DETCs in control and K14ΔNLef1 mice. As already qualitatively shown in the original version, the distribution of LCs and DETC reflects confinement to interscale IFE in both genotypes (revised **Fig. 6G,H**). Regarding the numbers of LCs per area, however, we noted a specific reduction in the interscale compartment of K14ΔNLef1 mice (revised **Fig. S5C**). Though the underlying causes remain subject of future investigations, it is tempting to speculate that LCs are perhaps unable to compete with the ectopic MCs residing in the interscale region of K14ΔNLef1 mice, consequently decreasing LC numbers.

More importantly, what are the functional/physiological consequences to MC biology now that they localize to the interscale region? Do they change their ability to perform critical MC functions? Do they increase their “immune- surveillance” functions? Laser capture experiments/Spatial transcriptomics/utilization of alternate marker expression with subsequent sorting/sequencing could be used to tease some of the questions apart.
Thus, while this manuscript is nicely written and discusses an interesting area of skin biology, mechanistic/functional experiments would be needed to be generate a manuscript of broader interest to the Development readership.

We agree with this reviewer that there are various new and exciting questions arising from our findings, and we are eager to investigate these in the mid-term using suited genetically traceable models. Within the scope possible for this revision, we have started to address the interesting question of how relocation of MCs to interscale affects their function. We explored MC functionality in K14ΔNLef1 mice by means of melanin production -a hallmark of MC differentiation and function- and related morphometric parameters (see also our response to point 1). CellProfiler-assisted automated morphometric analyses of interscale MCs in K14ΔNLef1 mice revealed an increased cell size (area) compared to WT MCs in interscale (revised **Fig. S6A**), as well as a pronounced dendritic morphology and major axis length comparable to control MCs (revised **Fig. S6B-F**). This is in line with robust melanin production of MCs in K14ΔNLef1 interscale regions, alike their wild-type counterparts (revised **Fig. S6G**). Apoptosis as assessed by cleaved Caspase3 immunohistochemistry did not reveal altered viability of MCs of K14ΔNLef1 mice (revised **Fig. S6H**). Together, these data indicate that repression of Wnt signaling in the epidermis causes significant repositioning of MCs to interscale IFE, whereby these MCs remain their ability to differentiate and produce melanin pigment (revised **discussion**, **last paragraph**, page 10). Future work beyond the scope of this manuscript is necessary to address potential differences regarding immune-surveillance functions of interscale MCs.

© 2022. Published by The Company of Biologists under the terms of the Creative Commons Attribution License (https://creativecommons.org/licenses/by/4.0/).

Second decision letter

MS ID#: DEVELOP/2021/200154

MS TITLE: Lrig1 and Wnt dependent niches dictate segregation of resident immune cells and melanocytes in murine tail epidermis

AUTHORS: Susanne C. Baess, Ann-Kathrin Burkhart, Sabrina Cappello, Annika Graband, Kristin Sere, Martin Zenke, Catherin Niemann, and Sandra Iden

I have now received all the referees reports on the above manuscript, and have reached a decision. The referees' comments are appended below, or you can access them online: please go to BenchPress and click on the 'Manuscripts with Decisions' queue in the Author Area.

As you will see, both referees are satisfied with your revised manuscript, and pending final revisions, we will be happy to publish the article in Development. Please could we ask you just to address the remaining concerns of referee 2, both in terms of providing quantitation and ensuring we have high quality images for publication? I hope these final changes should be straightforward but please let us know if you have any questions or concerns.

Unfortunately the article can not now be accepted for our Special Issue on the immune system as the deadline has passed, but we will be pleased to publish it in a 'normal' issue of the journal.

Reviewer 1

Advance summary and potential significance to field

I am happy that the revised manuscript addresses my comments. No further major concerns.

Comments for the author

I am happy that the revised manuscript addresses my comments. No further major concerns.

Reviewer 2

Advance summary and potential significance to field

Reviewer response to Baess et al., (Development, full revision response)

From this round of revisions, the authors have sufficiently addressed our concerns and have improved their manuscript. A few minor follow up points:

- Fig S2A: quantification of MCs should be accompanied with IF images actually showing MCs in the Ctrl and Id2KO (in addition to just K31 as is currently shown).
- Fig 3C appears very dark: the contrast of the immune cells should be adjusted so that the immune cells can be clearly seen in the Ctrl animals.
- Several panels (such as F1A, F2A, F3C, F6B) appear to have compressed(?) images which need to be fixed for final publication.

Comments for the author

From this round of revisions, the authors have sufficiently addressed our concerns and have improved their manuscript. A few minor follow up points:

- Fig S2A: quantification of MCs should be accompanied with IF images actually showing MCs in the Ctrl and Id2KO (in addition to just K31 as is currently shown).
- Fig 3C appears very dark: the contrast of the immune cells should be adjusted so that the immune cells can be clearly seen in the Ctrl animals.
- Several panels (such as F1A, F2A, F3C, F6B) appear to have compressed(?) images which need to be fixed for final publication.

© 2022. Published by The Company of Biologists under the terms of the Creative Commons Attribution License (https://creativecommons.org/licenses/by/4.0/).

Second revision

Author response to reviewers' comments

Reviewer 2 Comments for the Author:
From this round of revisions, the authors have sufficiently addressed our concerns and have improved their manuscript. A few minor follow up points:

- Fig S2A: quantification of MCs should be accompanied with IF images actually showing MCs in the Ctrl and Id2KO (in addition to just K31 as is currently shown).

  As stated in the legend of our first revision, the quantitation shown in Figure S2B relates to main Figure 3A. Hence, the accompanying IF images for S2B (and 3B) showing MCs in the Ctrl and *Id2*KO are to be found in main Figure 3A.

- Fig 3C appears very dark: the contrast of the immune cells should be adjusted so that the immune cells can be clearly seen in the Ctrl animals.

  We thank the reviewer for this comment, and agree with the insufficient brightness. We have now revised Figure 3 (A and C) coherent with the journal guidelines, to show an improved visualization of immune cells in the Control animals (revised Figure 3).

- Several panels (such as F1A, F2A, F3C, F6B) appear to have compressed(?) images which need to be fixed for final publication.

  Initially, we had uploaded high resolution images for the revision, requested as per the journal guidelines. Thereafter the *Development* office approached us and asked for a peer-review file sized less than 20 MB in total. This reviewer probably evaluated these files that are indeed of reduced quality, while the figure source files are of much better and publication-ready quality. We will send the *Development* office a separate download link to the high quality figure files (we tried uploading through the online submission system but learned about a 250 MB limit during the procedure).

Third decision letter

MS ID#: DEVELOP/2021/200154

MS TITLE: Lrig1 and Wnt dependent niches dictate segregation of resident immune cells and melanocytes in murine tail epidermis

AUTHORS: Susanne C. Baess, Ann-Kathrin Burkhart, Sabrina Cappello, Annika Graband, Kristin Sere, Martin Zenke, Catherin Niemann, and Sandra Iden
ARTICLE TYPE: Research Article

I am happy to tell you that your manuscript has been accepted for publication in Development, pending our standard ethics checks.

© 2022. Published by The Company of Biologists under the terms of the Creative Commons Attribution License (https://creativecommons.org/licenses/by/4.0/).
